# Vagal Flexibility during Exercise: Impact of Training, Stress, Anthropometric Measures, and Gender

**DOI:** 10.1155/2020/6387839

**Published:** 2020-10-05

**Authors:** Perciliany Martins de Souza, Nacha Samadi Andrade Rosário, Kelerson Mauro de Castro Pinto, Poliana Elisa Assunção, Fernando Luiz Pereira de Oliveira, Eduardo Bearzoti, Gabriela Guerra Leal Souza

**Affiliations:** ^1^Department of Biological Sciences, Federal University of Ouro Preto, Ouro Preto, Brazil; ^2^Sports Center, Federal University of Ouro Preto, Ouro Preto, Brazil; ^3^Department of Statistics, Federal University of Ouro Preto, Ouro Preto, Brazil

## Abstract

We evaluated the effect of physical training, stress, anthropometric measures, and gender upon the reactivity and recovery of the heart rate variability (HRV) during a cardiorespiratory test. Professors (*N* = 54) were evaluated using the following: physical training: time, frequency, and length of physical exercise; resting heart rate (HR); maximum HR; and recovery HR; stress: stress symptoms, work stress, vital events, and perceived stress; anthropometric measures: body mass index, waist circumference (WC), waist-hip ratio (WHR), and fat percentage (FP); and HRV before, during, and after the test. The HRV decreased during and increased after the test. Increased recovery HR was associated with the decreased vagal output during the test, and decreased recovery HR was associated with the increased posttest vagal input. The higher the work control and stress symptoms of men and the higher the perceived stress for both genders, the lower the vagal output during the test. The lower stress symptom and work control of men and the lower work demand of women were associated with the posttest vagal increase. The increased WC and decreased WHR of men were associated with the lower vagal output during the test and the lower posttest vagal increase. The lower FP also was associated with the greater recovery.

## 1. Introduction

The cardiac autonomic changes, including the parasympathetic (vagal) decrease and sympathetic increase, inherent to physical exercise, create a situation which favors ventricular ectopic activities, which might culminate in cardiac arrest or sudden death [1]. Therefore, Albert et al. [2] proposed the model of “window of exposure” to cardiovascular risks. According to those authors, due to the unfavorable autonomic state during a few minutes after physical exercise, physical activity increases exposure to cardiovascular risks, which only ends when the autonomic parameters return to the resting values. This issue is related to the responsive adaptation of vagal activity, which in turn might be regarded as a kind of flexibility. The concept of vagal flexibility has been used to explain a high autonomic reactivity to a specific stimulus and the return of this response to baseline values quickly afterwards [3]. Such ability can best be observed by studying the autonomic nervous system, for example, by analyzing the heart rate variability (HRV).

The HRV is a noninvasive phenomenon that reflects the continuous RR interval oscillations due to the effect of the sympathetic and parasympathetic nerve activity upon the sinoatrial node and has been used to assess changes in the autonomic function [4]. Although resting HRV is well studied, its assessment during exercise is not yet fully understood, mainly due to inconsistent results (e.g., lack of vagal withdrawal during a physical test) that might result from the variety of methods and exercise protocols used (maximal and submaximal effort) [5, 6] or the fitness level, endurance, body composition, or sex differences [7–9].

Among the possible effects on HRV, individuals with low fitness levels noticeably have a stronger sympathetic response during physical exercise. The resting HRV of those individuals is lower, and the “window of exposure” to cardiovascular risks is larger; that is, these individuals take longer to recover and consequently have less vagal flexibility. During exercise, intensity may produce different responses, with variations depending on the level of effort and sex [8].

The HRV might also be affected by psychosocial stress, which is a key factor in the development of cardiovascular diseases which also affects the autonomic recovery. Some jobs are notable for being psychosocial stressors, including teaching [10]. Seegers and van Elderen [11] suggested that work stress results from the perception that the work tasks exceed the personal capacity of the professionals to meet them. Such capacities are individual because some people, when exposed to specific demands, may react positively and dedicate themselves to meet them, whereas others might perceive the demands as threatening, thereby experiencing negative stress in such situations. Self-perceived stressful work situations [12, 13] (high demand and low control) might result in a high level of stress [14]. Individual characteristics, gender, and social support might minimize or exacerbate stress reactions and, in turn, the diseases caused by stress [11].

Finally, epidemiological studies have shown a relationship between cardiovascular risk factors and anthropometric variables—increased body mass, body mass index (BMI), waist circumference (WC), hip circumference (HC), and waist-hip ratio (WHR). A study from our group showed that age, WC, visceral fat, and systolic blood pressure are negatively associated with HRV vagal components [15]. Anthropometric changes were associated with low fitness levels, low levels of vagal activity, or high levels of stress, all of which are related to cardiac morbidity [15–18].

Thus, our aim was to investigate whether physical training level, stress, anthropometric measures, and gender affect the reactivity and recovery of HRV vagal components during a submaximal cardiorespiratory test in university professors. This study presents some relevant points: the sample homogeneity (Brazilian professors of a public university) and multiple variables collected on the same sample (vagal HRV components, physical training, stress, and anthropometric measures). We choose this sample because there are few studies with this group, and in general, they are a sample with high levels of stress, obesity, and sedentarism.

## 2. Materials and Methods

### 2.1. Participants

A total of 54 university professors, including 30 men and 24 women (42.59 ± 9.20 years), took part in this study. The inclusion criteria were aged from 25 to 65 years; being a tenured university professor; and not having a diagnosis of psychiatric, psychological, cardiac, or musculoskeletal disease. The study was approved by the Ethics Committee of the Federal University of Ouro Preto. All participants signed the informed consent form and followed the following recommendations before the tests: not performing intense physical exercises in the previous 48 hours; not consuming alcohol in the previous 24 hours; and not ingesting caffeine in the previous 12 hours.

### 2.2. Cardiorespiratory Test

We have performed the Fred and Kash test (1968), which is a submaximal test that measures the aerobic capacity, which consisted of stepping up onto a 30 cm high bench 24 times per minute (metronome at 98 beats per minute) for 3 minutes [19]. We used the HR 1 minute after the end of the test as an index of cardiorespiratory fitness as recommended by the test protocol. Before the test, the volunteers were instructed to perform the step according to the rhythm helped on the metronome.

### 2.3. Questionnaires

Lipp's Stress Symptom Inventory for Adults (LSSI) [14] is a questionnaire that provides an objective measure of the stress symptomatology experienced in a given time period: alarm phase (previous 24 hours), resistance phase (previous week), and exhaustion phase (previous month).

The work stress questionnaire [12, 13] is a questionnaire that measures the following dimensions: (1) work demand, which refers to psychological pressures, either quantitative (e.g., work time and speed) or qualitative (e.g., conflicts between contradictory demands); (2) work control, which is the possibility of the worker using his intellectual abilities to perform his work, as well as having sufficient authority to make decisions on how to perform it; and (3) work social support, which is the level of social interaction that exists at work, both with coworkers and supervisors.

The Vital Events Scale [20] is a scale assessing which vital events people have experienced in the previous year in the following categories: work, loss of social support, family, changes in environment, personal difficulties, and finances. The Perceived Stress Questionnaire (PSQ) [21] measures the degree to which individuals perceived situations as stressful during the previous month. Volunteers who were physically active were asked about the type exercise, the length (years of practice), the weekly frequency (days), and the duration (minutes) of each workout session. These questions were constructed by the study team.

### 2.4. Anthropometric Measures

Body mass was assessed using a Glicomed® scale, model BALGL3C, with a capacity of 150 kg and accuracy of 100 g. Height was measured using a metal stadiometer attached to the wall that was accurate to 1 mm. The procedures adopted by Fontanive et al. [22] were followed in both cases. BMI was calculated using the formula: body mass/height^2^ [23]. WC and HC were also measured, and WHR was calculated. In order to calculate the percentage of body fat using the SIRI formula [fat percentage = (4.95/density corporal) − 4.5 × 100] [24] we have measured three skinfolds (subscapular, suprailiac, and thigh) in women and three skinfolds (triceps, suprailiac, and abdominal) in men [25]. For this purpose, we have calculated in advance the body density [body density of women = 1.1665 − (0.0706∗LOG (*Σ* of skinfolds)] or [body density of men = 1.1714 − 0.0671∗LOG (*Σ* of skinfolds)].

### 2.5. Heart Rate Variability

A HR monitor Polar RS800CX (Polar Electro Oy, Kempele, Finland) was used to record the HRV. The time units were set at 1 millisecond, and the RR interval samples were collected at a sampling frequency of 1000 Hz [4]. The data collected using the HR monitor was transferred to a computer using the software *Polar ProTrainer 5*, through an interface with an infrared device. Then, that database was exported as text, and the RR interval signals were processed in order to calculate the HRV parameters using the Kubios HRV Analysis software (MATLAB, version 2 beta, Kuopio, Finland). We have used the root mean square of the successive differences (RMSSD) in RR intervals (time analysis) and the standard deviation data of the instantaneous beat-to-beat variability (SD1) (Poincaré plot analysis) because both components represent the parasympathetic activity in the ANS and are not so influenced by respiratory rate changes [4, 7].

During all phases of the experiment (rest, exercise, and recovery), RR interval series were visually inspected for artefacts and automatic corrected using medium Kubios artefact correction, which replaces detected artefact beats using cubic spline interpolation. The “medium” correction level identifies all RR intervals that are larger/smaller than 0.25 seconds compared to the local average. The correction is made by replacing the identified artefacts with interpolated values using a cubic spline interpolation.

The components used in the HRV assessment were 3 minutes at rest, 3 minutes during the submaximal cardiorespiratory test, and 3 minutes in the posttest recovery time. The reactivity index, which is given by the HRV during the test, HRV during rest (Reac (exe-rest)), and the recovery index, which is given by the HRV during recovery, HRV during the test (Rec (rec-exe)), were calculated using those data. As we are interested in the vagal flexibility during the exercise, we decided to use the reactivity and recovery indexes, as recommended by Balzarotti et al. [26] and Laborde et al. [27].

### 2.6. Procedures

The volunteers were invited by letters, and after acceptance, they scheduled a time for the experiments in the laboratory and received the recommendations for the tests. In the day of the test, the volunteer read and signed the informed consent form. After, they had the anthropometric variables measured, and then, they filled out the Cardiovascular Risk Stratification Questionnaire and Physical Activity Readiness Questionnaire (both to ensure the safety of the volunteers during the cardiorespiratory test) and the stress questionnaires. Next, the researcher cleaned the participant's skin with alcohol 70% and put the electrode belt following Polar's guidelines, tightly but comfortably just below the chest muscles. Then, the participants performed the cardiorespiratory test (3-minutes rest–3-minute exercise–3-minute recovery). At the end of the experiment session, the electrodes were removed, and the participants were thanked and debriefed.

### 2.7. Statistical Analysis

Initially, data normality was tested using the Shapiro-Wilk test. The parasympathetic activation/inhibition behavior before, during, and after the physical test and the gender effects were evaluated employing the repeated-measures analysis of variance (ANOVA), with time (rest, exercise, and recovery) being the within-group variable and gender (man or woman) as the between-group variable, with the Greenhouse–Geisser correction. The Tukey post hoc test was run for the significant results.

The stepwise regression analysis with backward elimination of variables was used to describe the effect of the variables of interest (level of training, anthropometric measures, and stress), gender, and the interaction between them and gender on the reactivity and recovery indices [28]. For each class of variables (for example, cardiorespiratory fitness variables), the initial model used in this method used all variables and a dummy variable [28] to discriminate genders and compared the products of this dummy variable with each variable. The dummy variable was defined as being 1 for men and 0 for women. Residual analysis to was done to validate the models.

For all tests performed, the level of significance adopted was 0.05.

## 3. Results

### 3.1. Gender Differences in the Level of Physical Training, Stress, Anthropometry, and HRV Vagal Components

No significant differences were found as a function of gender regarding age (men = 38.5 ± 9.35 years and women = 43.9 ± 8.93 years, *p* = 0.19) and teaching time at the institution (men = 6.79 ± 4.5 years and women = 7 ± 4.49 years, *p* = 0.28). Women had higher fat percentage (*p* = 0.000002), maximum HR (*p* = 0.0001), recovery HR (*p* = 0.001), perceived stress (*p* = 0.001), and stress symptoms in the previous week (*p* = 0.02) and previous month (*p* = 0.004) and lower waist circumference (*p* = 0.000004) and waist-hip ratio (*p* = 0.000001) than men. The Table 1 presents all the differences between the sexes for the variables of interest.

### 3.2. Assessment of the Relationship between the Physical Training, Sex, and Vagal Flexibility during the Cardiorespiratory Test

The ANOVA for RMSSD showed a major effect of time (*F*_(2.104)=_35.61, *p* < 0.0001, *ε* = 0.86). The posttest showed a decrease in RMSSD from rest to exercise and an increase from exercise to recovery (*p* = 0.0001 for both comparisons). No significant effect of gender (*F*_(1.52)=_1.691, *p* = 0.2) or from the interaction between time and gender (*F*_(2.104)=_1.04, *p* = 0.3, *ε* = 0.86) was found. The ANOVA for SD1 showed a major effect of time (*F*_(2.104)=_43.87, *p* < 0.0001, *ε* = 0.86). The posttest showed a reduction in SD1 from rest to exercise and an increase from exercise to recovery (*p* = 0.0001 for both comparisons). No major effect of gender (*F*_(1.52)=_1.63, *p* = 0.2) or from the interaction between time and gender (*F*_(2.104)=_0.846, *p* = 0.4, *ε* = 0.86) was observed.

Regression model 1 was carried out to examine the relationship between the physical training variables and each vagal reactivity and recovery indices. No gender effect occurred at any time. Higher recovery and resting HRs were associated with the significant lower vagal withdrawal during the test (higher RMSSD exe-rest, according to the positive signs of the regression coefficient in Table 2). Lower recovery HR was associated with higher vagal inputs after the end of the test (increased RMSSD rec-exe and SD1 rec-exe, since the regression estimates were negative, see Table 2).

Regression model 2 was employed to examine the relationship between stress variables and each vagal reactivity and recovery indices. The RMSSD and SD1 reactivity were only affected in men, and all significant stress variables that affected such behavior were equal for both analyzed HRV components. Thus, higher work control corresponds to higher perceived stress, stress symptoms in the previous week, and lower vagal withdrawal during test (higher RMSSD exe-rest and SD1 exe-rest). Conversely, few stress symptoms in the previous month corresponded to lower vagal withdrawal (higher RMSSD and SD1 exe-rest). Less control of the work activities by men was associated with the increase in vagal input after the test (higher RMSSD and SD1 rec-exe). For women, lower work demands were associated with the increase in vagal input after the test (SD1 rec-exe; Table 2).

Regression model 3 was used to examine the relationship between anthropometric variables and each vagal reactivity and recovery indices. An increase in the WC of men would explain the significantly lower vagal withdrawal during the test (higher RMSSD exe-rest and SD1 exe-rest indicators) and the lower vagal increase during recovery (lower RMSSD rec-exe indicator). A decrease in the WHR of men would also explain the lower vagal withdrawal during the test (higher RMSSD exe-rest and SD1 exe-rest indicators), and its increase would explain a weaker parasympathetic response during recovery (lower RMSSD rec-exe indicator and SD1 rec-exe). The recovery of the SD1 component (lower SD1 rec-exe indicator) might also result from a lower BMI, regardless of gender (Table 2).

## 4. Discussion

HRV represents a way of studying the response of the autonomic nervous system to exercise. As reported by Tulppo et al. [29] and Michael et al. [7], vagal withdrawal occurs already in the initial seconds of the exercise and is influenced both by exercise length and intensity. Parasympathetic recovery also restarts in the first moments after the end of the exercise; although in contrast to the findings of Michael et al. [7], the present study showed that 3 minutes was enough for a return to the RMSSD and SD1 resting levels. Some authors have shown that individuals who recover more rapidly from physical exercise have a small window of exposure to cardiovascular complications [30]. Our results indicate that our sample of professors had a small window of exposure and adequately recovered from the cardiorespiratory test.

The Kash bank test used here is considered a submaximal test to assess cardiorespiratory fitness. However, when analyzing the maximum HR of the test in the present sample, it is observed that the percentage in relation to the average of the maximum estimated HR (estimated HR = 220 − age) [31] for women was 66 to 102%, differing significantly from the percentage of men, which was 58 to 98% of the estimated maximum HR (*p* = 0.0003). These percentages, in addition to indicating a difference between the sexes in the maximum HR reached, also showed that the test, despite having been classified and validated in the literature as submaximal, for the present sample promoted a very large variation, even exceeding the maximum values of estimated HR.

We choose RMMS and SD1 parameters because RMSSD is a classic component that reflects HRV vaguely mediated and is relatively free from respiratory influences, compared to high-frequency parameters [32]. The SD1 component is a parameter that has been explored more recently and seems to be the most suitable for studies during a physical effort where the HRV linearity is broken [33].

After observing the changes in HRV vagal components resulting from exercise, we sought to identify those that contributed to the occurrence of this vagal flexibility using regression models. Regarding the level of training, we observed that higher recovery and resting HRs were associated with a lower vagal withdrawal during the test. Similarly, lower recovery HRs were associated with a higher vagal input after the end of the test; these changes occurred without any gender effect. Confirming our findings, Javorka et al. [34] found a correlation between the recovery values 1 minute after the end of the physical exercise and HRV components. Conversely, our findings also suggest a key role of recovery HR, perhaps because it is also an important component that affects the “window of exposure” to cardiovascular risk, as suggested by Albert et al. [2]. This result contrasts with previous reports indicating that only the resting HR was a key predictor of physical fitness and vagal flexibility [30, 35].

Regarding the recovery HR measured in the first postexercise minute, it is suggested that it is an efficient way of assessing cardiac vagal reactivation, since after the end of physical exercise, the vagus nerve begins to activate the sinoatrial node again and promotes HR decrease [36, 37]. In other words, in relation to the behavior of the cardiovascular control mechanisms postexercise, the literature has shown that when the input coming from the central command and the mechanoreceptors present in the skeletal muscles cease, the vagus nerve is reactivated and produces the fast decrease in HR [38, 39]. Cole et al. [40] also point out that the late decrease in HR during the first minute after exercise is a powerful predictor of general mortality, regardless of workload, presence of myocardial perfusion diseases, and changes in HR during exercise.

Our findings about the stress variables showed that higher control of work activities, higher perceived stress, and greater stress symptoms during the previous week were associated in men with lower vagal withdrawal during the test. For men, fewer stress symptoms during the previous month was also associated with lower vagal withdrawal. Decreased control of work activities was associated in men with increased vagal input after the test. Finally, a decrease in the work demand in women was associated with increased vagal input after the test. Further developing the concept of the Michigan model, our results suggest that increased control of work activities might require more sacrifice from individuals, that a low work demand is beneficial to their health, and that personal characteristics could contribute to lower perceived stress and possibly to fewer stress symptoms [11, 12, 14, 21].

The anthropometric measures that affected vagal flexibility were the following: increased WC for men, which was associated with lower vagal withdrawal during the test; and lower vagal increase during recovery, representing an adaptive physiological response to exercise. On the other hand, the decrease in WHR was associated in men with the decreased vagal withdrawal during test and the increased vagal input during the recovery. This is a much unexpected result because WC and WHR, being similar measures, should behave similarly in response to exercise. Finally, lower BMI was associated with higher SD1 recovery, regardless of the gender of the participants. Some authors showed associations between an increase in the anthropometric measures and a decrease in the vagal components of HRV, during the resting period [15, 18]. In the present study, this result was replicated and expanded, thus showing that an increase in anthropometric measures might indeed affect adversely the vagal flexibility during the test. Accordingly, the results showed that in addition to the cardiovascular involvement resulting from the increase in those measurements, they also have a deleterious effect on the “window of exposure” to cardiovascular risks [30].

This study has some limitations: (i) the sample included only university professors; (ii) the physical text was a submaximal test; (iii) the sample was relatively small; and (iv) volunteers were not at high levels of stress and severe anthropometric negative changes.

## 5. Conclusions

We concluded that the submaximal cardiorespiratory test was able to promote changes in the vagal components (reduction during the exercise and increase after it), regardless of gender. We noted that vagal flexibility was affected by the resting and recovery HR, control and demand of work activities, perceived stress, stress symptoms during the previous week and month, WC, WHR, and BMI. From this perspective, we believe that the results herein presented could help in the prescription of physical exercises, so that the understanding of the factors that affect the vagal flexibility to the exercise would be critical to guide the exercise and care with the practitioners of different types of exercise. In addition, it is also an important factor to be considered in order to obtain better results, either in the physical performance or in the quality of life.

## Figures and Tables

**Table 1:** Descriptive variables and differences between men and women.

Variable	Men (*N* = 28)		Women (*N* = 22)		*p* value
	Mean ± SD/median (P25/P75)		Mean ± SD/median (P25–P75)		
Level of physical training variables					
Exercise frequency (days)	2	0/3.5	2	0/3	0.28
Workout duration (minutes per workout)	60	0/90	45	0/50	0.09
Exercise time (years)	0.66	0/4.5	0.50	0/2	0.59
Resting HR (bpm)	71.76	±10.88	76.11	±9.14	0.13
Maximum HR (bpm)	137.42	±15.66	154.68	±13.84	0.0001^∗^
Recovery HR—1 minute (bpm)	91.21	±16.92	110.22	±21.92	0.001^∗^
Stress variables					
Work stress					
Demand	14.50	12.5/15.5	15	13.5/18	0.17
Control	20	19/21	20	19/21	0.32
Social support	18.07	±3.31	17.45	±3.54	0.52
Perceived stress	21.07	±8.77	29.09	±7.17	0.001^∗^
Vital events	2	2/3.5	3	2/5	0.22
Stress symptoms					
24 hours	1	0/2	2	1/3	0.09
Previous week	1.5	0/3.5	3	1/5	0.02^∗^
Previous month	2	1/3.5	4.5	2.5/6.5	0.004^∗^
Anthropometric variables					
BMI (kg/m^2^)	25.56	±3.59	23.78	±3.18	0.07
WC (cm)	87.4	±9.0	74.9	±7.6	0.000004^∗^
WHR	0.8	±0.1	0.7	±0.1	0.000001^∗^
Fat percentage (%)	20.1	±5.8	27.9	±3.6	0.000002^∗^
HRV variables					
RMSSD exe-rest	-18.52	-32.90/-12.18	-18.0	26.0/-11.5	0.79
SD1 exe-rest	-15.33	-20.98/-1.03	-14.53	-18.85/-10.69	0.93
RMSSD rec-exe	24.02	±23.23	15.26	±18.42	0.15
SD1 rec-exe	17.18	±13.21	11.94	±12.34	0.15

kg: kilogram; BMI: body mass index; m: meters; kg/m^2^: kilogram per square meter; WC: waist circumference; cm: centimeters; WHR: waist-hip ratio; HR: heart rate; bpm: beats per minute; RMSSD: root mean square of the successive differences between RR intervals; SD1: standard deviation 1; exe-rest: exercise minus rest (reactivity); rec-exe: recovery minus exercise (recovery); ^∗^indicative of a significant difference between genders (<0.05).

**Table 2:** Results of the regression analysis for the relationship between anthropometric, physical training, and stress variables, and the HRV indices that assess reactivity and recovery.

	Variable	Model 1: physical training	*β*	*R* ^2^	Model 2: stress	*β*	*R* ^2^	Model 3: anthropometry	*β*	*R* ^2^
Reactivity	RMSSD exe-rest	Recovery HR	0.3968	0.1159	*Z*	-122.0918	0.4413	Waist circumference∗*Z*	1.9409	0.1904
					Control∗*Z*	6.4640		WHR∗*Z*	-199.0476	
					Perceived stress	1.2391				
					Previous week symptoms∗*Z*	12.4345				
					Previous month symptoms∗*Z*	-12.1427				
	SD1 exe-rest				*Z*	-81.0581	0.4158	Waist circumference∗*Z*	1.0737	0.1300
					Control∗*Z*	4.3806		WHR∗*Z*	-110.2779	
					Perceived stress	0.8069				
					Previous week symptoms∗*Z*	6.13				
					Previous month symptoms∗*Z*	-8.4507				
Recovery										
	RMSSD rec-exe	Recovery HR	-0.6498	0.4154	*Z*	72.2213	0.2461	Waist circumference∗*Z*	-1.7238	0.0839
					Previous week symptoms∗*Z*	-4.4386		WHR∗*Z*	181.2200	
					Previous month symptoms∗*Z*	6.5614				
					Demand	3.1534				
					Demand∗*Z*	-3.9841				
	SD1 rec-exe	Recovery HR	-0.3757	0.3811	*Z*	99.8430	0.3359	Waist circumference∗*Z*	-0.1622	0.2851
					Control∗*Z*	-2.8425		WHR	76.8599	
					Previous week symptoms∗*Z*	-5.8383		Fat percentage	-1.2513	
					Previous month symptoms∗*Z*	4.4578				
					Demand	2.0380				
					Demand∗*Z*	-2.3898				

WHR: waist-hip ratio; % fat: fat percentage; HR: heart rate; RMSSD: root mean square of the successive differences between RR intervals; SD1: standard deviation 1; SD2: standard deviation 2; exe-rest: exercise minus rest (reactivity); rec-exe: recovery minus exercise (recovery); *Z*: interactions with gender.

## Data Availability

We are willing to make all our data available, if necessary.
